# Laminin Adsorption and Adhesion of Neurons and Glial Cells on Carbon Implanted Titania Nanotube Scaffolds for Neural Implant Applications

**DOI:** 10.3390/nano12213858

**Published:** 2022-11-01

**Authors:** Jan Frenzel, Astrid Kupferer, Mareike Zink, Stefan G. Mayr

**Affiliations:** 1 Leibniz Institute of Surface Engineering (IOM), 04318 Leipzig, Germany; 2 Division of Surface Physics, Faculty of Physics and Earth Sciences, Leipzig University, 04103 Leipzig, Germany; 3 Research Group Biotechnology and Biomedicine, Faculty of Physics and Earth Sciences, Leipzig University, 04103 Leipzig, Germany

**Keywords:** titania nanotubes, low-energy ion implantation, laminin adsorption, neurons and glial cell response, biocompatibility, neural implant

## Abstract

Interfacing neurons persistently to conductive matter constitutes one of the key challenges when designing brain-machine interfaces such as neuroelectrodes or retinal implants. Novel materials approaches that prevent occurrence of loss of long-term adhesion, rejection reactions, and glial scarring are highly desirable. Ion doped titania nanotube scaffolds are a promising material to fulfill all these requirements while revealing sufficient electrical conductivity, and are scrutinized in the present study regarding their neuron–material interface. Adsorption of laminin, an essential extracellular matrix protein of the brain, is comprehensively analyzed. The implantation-dependent decline in laminin adsorption is revealed by employing surface characteristics such as nanotube diameter, ζ-potential, and surface free energy. Moreover, the viability of U87-MG glial cells and SH-SY5Y neurons after one and four days are investigated, as well as the material’s cytotoxicity. The higher conductivity related to carbon implantation does not affect the viability of neurons, although it impedes glial cell proliferation. This gives rise to novel titania nanotube based implant materials with long-term stability, and could reduce undesirable glial scarring.

## 1. Introduction

Driven by a rapidly aging society, neurodegenerative diseases pose an ascending medical challenge due to the steadily increasing influx of patients. For example, the global median age increased by 11.68% between 1990 and 2015, while the number of Parkinson’s disease patients doubled [1,2]. Meanwhile, the World Health Organization estimates that neurodegenerative diseases affect about one billion people worldwide [3].

Targeting such diseases by enlightening neural network dynamics to gain a deeper insight into communication between neurons, along with microelectrode arrays (MEAs) for in vitro screening, appears to be a promising strategy [4,5]. Through multiple thin electrodes, electrical potential differences can be sensed from a few neurons up to a large cell population or even on the scale of brain tissue. Additionally, MEAs utilized as a lab-on-a-chip device can be coupled to an optical system [6,7]. In this way, alterations in cell and tissue structures can be assessed by fluorescence microscopy techniques when performing electrophysiological experiments. Furthermore, MEA and even other shapes of electrodes implanted in brains can be used as in vivo electrodes for deep brain stimulation (DBS) to excite the neurons of patients who suffer from, e.g., Parkinson’s disease [8,9]. This technique offers a promising approach for a symptomatic treatment which leads to an exceptional enhancement of patients’ quality of life [10].

However, the interconnection between cells or tissue and the electrode materials mechanically is often mismatched [11,12]. Moreover, implant micromotion, foreign-body response, and enhanced reaction of microglia on in vivo implanted electrode materials result in impaired electric signal transmission [13]. Thus, a function breakdown of the implants’ intended use is predicted [14,15]. Consequently, the electrode materials require reimplantation in a different location after several years or sometimes even after a few months, resulting in additional neuronal tissue damage [16,17].

In order to reduce mechanical mismatches and to enhance the interconnection between cells/tissues and electrode materials, both the choice of suitable biocompatible materials as well as further strategies regarding surface structuring are crucial. Hence, a substitution of obsolete electrode materials made of stainless steel, tungsten, platinum, iridium oxide, or gold is anticipated [18,19,20,21,22]. Emendations have already been reported with electrode materials consisting of carbon, silicon, or composites such as platinized carbon fibers or silicon with a porous titanium nitride surface [23,24,25,26]. There is particular room for improvement with regard to long-term stability. Nowadays, soft electrode materials consisting of polymers such as PEDOT [27], polypyrrole [28], and polythiophene [29] can likewise be considered, as they significantly reduce rejection reactions. However, these materials are not as flexible [30], have poorer electrical properties, and exhibit reduced long-lasting mechanical behavior [31,32].

To tackle such problems, modified titanium and its oxide, titanium dioxide (TiO${}_{2}$), are the first line of materials chosen for implants, combining chemical stability, biocompatibility, and excellent corrosion resistance [33,34,35,36]. In order to provide optimized tissue integration, the implant surfaces are tailored in such a way that the adsorption of proteins of the extracellular matrix is promoted and cell adhesion is favored [33]. This can be realized, for instance, by physicochemical nanostructuring processes and culminates in distinct geometries [37,38,39,40]. In particular, titania nanotube scaffolds (TNSs) have great potential due to their high aspect ratios and their enormous surface [41]. Their morphology can be easily tailored by suitably adjusting the production parameters. I.e., electrochemical anodization and the connected self-organized nanotube growth can be fine-tuned in such a way that tube length, diameter, wall thickness, and surface morphology can be precisely regulated [42,43,44].

Such tailoring of TNSs enables long-term physiologic and organotypic culturing of adult neuronal tissues for several weeks [45,46]. In particular, the preservation of tissue structure and functionality of highly sensitive brain tissues combined with absence of degenerative effects in culture demonstrates the potential of TNSs as an electrode material for in vivo applications in the brain.

It is true that the mostly amorphous oxide layer of TNSs is semiconducting with a band gap of ∼3 eV, and thus tends to be ill-suited for electrically conductive implants [47]. However, this conductivity can be significantly increased by decorating TNSs with silver nanoparticles while maintaining high biocompatibility [48]. If precise control of the electrical conductivity is required, ion implantation is the method of choice due to the ability to freely adjust implantation fluences and energies. Recently, it has been demonstrated by Kupferer et al. [49] that even low carbon implantation fluences of 1 $\times \;10^{16}$ ions·cm${}^{-2}$ improve the conductivity of TNSs by at least three orders of magnitude, yielding a promising approach for the development of TNSs as brain–machine interfaces. However, shrinkage of the nanotubes and smoothing of the surface have been reported [49,50]. Because these parameters have been proven to affect interaction with soft matter, we investigate the biocompatibility of different low-energy low-fluence carbon implanted TNSs. The surfaces of the investigated TNSs are analyzed in terms of tube diameter shrinkage, surface free energy changes, and ζ-potential alterations. Subsequently, laminin adsorption is analyzed by various qualitative and quantitative methods. By linking the obtained results to the decreased tube diameter and the rising polar component of the surface free energy upon implantation, we elucidate the hindered protein adsorption. Furthermore, we explore the viability of glial cells and neurons as well as the toxicity of carbon implanted TNSs over two time periods by luminescence assay. Cell morphology is analyzed by fluorescence microscopy using antibody staining and corroborated by scanning electron microscope images of single cells. Thus, changes in cell viability, cytotoxicity, and morphology due to carbon implantation are connected to impeded protein adsorption as well as to shifts in electrical conductivity. These insights might contribute to the design of new electrode materials based on implanted TNSs to achieve long-term implant stability.

## 2. Materials and Methods

### 2.1. Titania Nanotube Scaffolds

Fabrication: Titania nanotube scaffolds (TNSs) were manufactured by electrochemical anodization in a self-organized growth process of a titanium foil (Advent Research Materials, England, Ltd., Witney, UK, 0.1 mm thickness, 99.6+ % purity) using a platinum mesh as cathode. First, the titanium foil was cleaned in an ultrasonic bath with isopropanol and water for 10 min each and subsequently dried under nitrogen flow. Afterwards, the foil was placed in a beaker containing ethylene glycol (CarlRoth GmbH and Co. KG, Karlsruhe, Germany), 2 vol% Milli-Q water and 0.3 wt% ammonium fluoride (MERCK EMSURE^®^ ACS, Darmstadt, Germany) at a distance of 45 mm from a platinum mesh. Anodization of the titanium foil was performed at 60 V for 60 min. Subsequently, the obtained layer was removed by ultrasonication. A second anodization in 0.6 wt% ammonium fluoride containing electrolyte for 6 min resulted in the final pore-like surface structure. To harden the TNSs, TNSs were immersed in ethylene glycol overnight. The day after, TNSs were cleaned with Milli-Q water in an ultrasonic bath for 10 min to remove anodization residues.

Carbon Implantation: Ion implantation was performed using an ion implanter (IMC-200, ion beam services S.A., Austin, TX, USA) with mass-separated C ions. Fluences of 8 $\times \;10^{14}\;\mathrm{ions}\cdot {\mathrm{cm}}^{-2}$ and 1 $\times \;10^{16}\;\mathrm{ions}\cdot {\mathrm{cm}}^{-2}$ were implanted into surface-near regions of the TNSs with an ion energy of 60 keV. For a fluence of 2 $\times \;10^{16}\;\mathrm{ions}\cdot {\mathrm{cm}}^{-2}$, first 1 $\times \;10^{16}\;\mathrm{ions}\cdot {\mathrm{cm}}^{-2}$ were implanted at an energy of 60 keV. Subsequently, the same fluence was implanted at a higher ion energy of 150 keV. During implantation, the samples were tilted by 7${}^{{}^{\circ}}$ to avoid channeling.

### 2.2. ζ-Potential

The ζ-potential of TNSs was characterized in solution for different pH values by the streaming potential method using an adjustable gap cell in a SurPass system (Anton Paar Germany GmbH, Ostfildern, Germany), as previously described in detail in Ref. [51]. For dissolved rhodamine-labeled laminin (LMN01, Cytoskeleton Inc., Denver, CO, USA) under neutral conditions, the ζ-potential and hydrodynamic radius of the protein was determined with dynamic light scattering experiments using a Malvern Zetasizer (Zetasizer Nano ZS, Malvern Panalytical GmbH, Kassel, Germany). A laminin concentration of 20 μg laminin per 1 mL Milli-Q water was analyzed.

### 2.3. Surface Free Energy

The surface free energy was analyzed using the sessile drop technique. Three droplets each of Milli-Q water, diidomethane, and ethylene glycol were applied to different locations on the examined TNSs using a contact angle meter (G2, Krüss GmbH, Hamburg, Germany). Images of the droplets were subsequently acquired, and six contact angles per droplet were measured using Fiji. Finally, surface free energy was determined using the Owens–Wendt–Rabel–Kaelble (OWRK) theory [52].

### 2.4. Protein Adsorption

Protein adsorption was investigated for four different protein concentrations, which were obtained by dissolving rhodamine-labeled laminin (0.1, 0.5, 1.0, 1.5 μg) in Milli-Q water (100 μL). Subsequently, the protein solutions were poured onto the TNSs at room temperature, resulting in an initial protein mass of 0.1, 0.5, 1.0, and 1.5 μg·cm${}^{-2}$, respectively. Qualitative adsorption behavior within 30 min was analyzed using an upright fluorescence microscope (ZEISS Axio Scope.A1; Carl Zeiss Microscopy GmbH) with 43 HE filter (Cat. No. 489043-9901-000, Carl Zeiss Microscopy GmbH) set at 10× and 40× magnification. Images of time-dependent protein adsorption were acquired alternately every 5 min at the droplet edge and at the TNSs surface using a camera (AxioCam ICm 1, Carl Zeiss Microscopy GmbH) and subsequently analyzed with ZEN lite software (Carl Zeiss Microscopy GmbH).

To quantitatively investigate protein adsorption, the unadsorbed protein solution was removed from the TNSs after 30 min and the laminin intensity was measured using a fluorescence plate reader (Synergy H1, BioTek Instruments Inc., USA). A standard curve of rhodamine-labeled laminin (0.0, 0.1, 0.2, 0.3, 0.5, 0.7, 1.0, and 1.5 μg) was used to evaluate these solutions in a black 96-well cell culture microplate (Cat. No. 655086, Greiner AG). A self-written Python script was used to evaluate the standard curve and link the measured fluorescence intensities of the TNSs to the protein amounts [53,54,55,56,57,58].

### 2.5. Cell Culture

To examine cell viability, cytotoxicity and morphology on TNSs, U87-MG Uppsala glioblastoma cells (Cat. No. 300367, CLS Cell Lines Service GmbH) and SH-SY5Y neuroblastoma cells (Cat. No. CRL-2266, ATCC LGC Standards GmbH) were used. Cells were cultured in 1:1 minimum essential medium (Cat. No. MEM-A; Capricorn Scientific GmbH) and Ham’s F-12 medium (Cat. No. 21765-029, Thermo Fisher Scientific Inc., USA), 10% fetal bovine serum (Cat. No. FBS-11A, Capricorn Scientific GmbH), and 1% penicillin/streptomycin (Cat. No. P0781-100ML, Sigma-Aldrich Chemie GmbH) at 37 ${}^{{}^{\circ}}$C and an atmosphere of 5% CO${}_{2}$. Cell culture medium was renewed every two days. Cells were passaged at a confluence of 80%. Accordingly, culture medium was removed and culture flask was rinsed with a solution containing phosphate-buffered saline (PBS, Cat. No. 18912014, Thermo Fisher Scientific Inc.), 0.025% trypsin-ethylenediaminetetraacetic acid, and 0.01% ethylenediaminetetraacetic acid solution (Cat. No. L2143, Biochrom GmbH). Subsequently, cells were incubated with solution until detachment, then transferred at a ratio of 1:10 for U87-MG and 1:5 for SH-SY5Y to a new culture flask.

To analyze the cells on the TNSs, TNSs were placed in ethanol and rinsed with Milli-Q water under a sterile hood. Subsequently, TNSs were dried at room temperature. Afterwards, TNSs were attached with carbon adhesive on the bottom of a 12-well cell culture plate (Cat. No. 665180, Greiner Bio-One GmbH) and a droplet of culture medium was placed on top of each TNS. In all cell experiments, cells were counted using a cell counter (EVE^™^, NanoEntek America, Inc.). For experiments with U87-MG cells, 2000 cells·cm${}^{-2}$ were added to each TNS for the one-day test and 1000 cells·cm${}^{-2}$ were placed on each TNS for the four-day test. To obtain the neuron-like behavior of SH-SY5Y cells, cells were differentiated for 72 h with staurosporine (25 nM, Cat. No. S5921-.1MG, Sigma-Aldrich Chemie GmbH). Then, 6000 cells·cm${}^{-2}$ were placed on each TNS for the one-day test and 9000 cells·cm${}^{-2}$ were added to each TNS for the four-day test.

Standard curves with different cell numbers were additionally generated for later investigation of cell counts by luminescence assay.

### 2.6. Cell Viability/Toxicity

To analyze the number of viable and dead cells, dead-cell protease activity of the cells on TNSs was studied using the luminescence signal of CytoTox-Glo^™^ assay (Cat. No. G9291, Promega Corp.). For preparation of lysis buffers and luminescence assay, the manufacturer’s protocol was followed.

Our experiments were performed by first removing the cell culture medium from the 12-well plate containing TNSs after the test period of one and four days, leaving 100 μL per well, and then tempering the plates to 22 ${}^{{}^{\circ}}$C for 30 min. Next, CytoTox-Glo^™^ cytotoxicity assay reagent (100 μL) was added to each well and the plate was shaken orbitally for 1 min at 807 cpm with a luminescence plate reader (Synergy H1, BioTek Instruments, Inc., USA). The stabilized luminescence signal was measured after 15 min, then correlated with a standard curve to obtain the amount of dead cells. Subsequently, lysis buffer (100 μL) per well was added and the plate was shaken again; 15 min later, the luminescence signal of each well was evaluated. The standard curves were analyzed to link the recorded intensities to the cell counts.

To measure the standard curves, the initial steps up to tempering were the same as described above. Subsequently, lysis buffer (100 μL) consisting of digitonin (13 μL) and assay buffer (2.75 mL) was added to each well in one row and assay buffer (100 μL) was added to each well of the other row. Then, the plate was shaken orbitally at 807 cpm for 2 min, and assay reagent (100 μL) was added to each well before the plate was shaken again for 1 min. After 15 min, the luminescence signals were measured. By subtracting the background signal from the signal with lysis buffer and linking it to the corresponding cell counts, a standard curve was generated. A self-written Python script was used to connect the standard curve to the measured intensities in order to extract the cell counts [53,54,55,56,57,58].

### 2.7. Fluorescent Staining

To study cell morphology, F-actin in the cytoskeleton was stained directly with fluorescent labeled phalloidin (Cat. No. 49409-10NMOL, Sigma Aldrich) at a dilution of 1:500 in methanol (Carl Roth GmbH and Co. KG) at room temperature. Therefore, cells on TNSs were fixed with 10% formalin solution (Cat. No. HT5011, Sigma Aldrich) for 15 min. Subsequently, cells were washed twice with wash buffer containing 0.05% Tween^®^20 (Cat. No. P9416, Sigma Aldrich) in 1× PBS solution (Cat. No. 18912014, Thermo Fisher Scientific Inc.). Next, cells were permeabilized with 0.1% Triton^®^ X-100 (Cat.No. 9002-93-1, Sigma-Aldrich Chemie GmbH) for 5 min. Subsequently, cells were washed twice with wash buffer and a blocking solution containing 1% bovine serum albumin (Cat.No. A2153-100G, Sigma-Aldrich Chemie GmbH) in 1% PBS was applied for 30 min. Afterwards, the staining solution was added and incubated for 60 min. This was followed by washing the cells in three washing steps, each lasting for 5–10 min. Finally, cells were covered with PBS to prevent them from drying out.

### 2.8. Image Processing and Cell Detection

For optical cell analysis, a fluorescence microscope was used with a filter set to 38 HE (Cat. No. 000000-1031-346, Carl Zeiss Microscopy GmbH) and a 5× magnification objective. The brightness and contrast of the captured microscope images of stained cells were first manually adjusted in the ZEN lite program (Carl Zeiss Microscopy GmbH) for all images. Afterwards, images were processed in Fiji as described by Weidt et al. [37], and an image threshold was defined. To detect individual cells, a minimum and maximum cell size was set, then an edge detection routine was used to detect single cells. In order to evaluate only entire cells, cells that were cut off at the image edge were excluded. Cell parameters such as cell size (*A*) and roundness ($4\cdot A\cdot \pi^{-1}\cdot l^{-2}$) were exported for each image. For the latter parameter, each cell was fitted with an ellipse and the major axis (*l*) was determined. Then, the parameters of all images were merged and evaluated with a Python script [55,56,57,58].

### 2.9. Atomic Force Microscope

Surface roughness was examined in ranges of 2 μm × 2 μm and 50 μm × 50 μm, respectively, with an atomic force microscope (Dimension Icon^®^, Veeco Instruments, Inc.). An OTESPA-R3 cantilever was used in tapping mode. Root mean square roughness ($R_{\mathrm{RMS}}$) was determined using Gwyddion [59].

### 2.10. Scanning Electron Microscope

A field emission scanning electron microscope (SEM, FEI Quanta 250 FEG, FEI Company) in high vacuum mode was employed to acquire images of the examined TNSs. The SEM was equipped with an Everhart–Thornley detector, and an acceleration voltage of 10 keV was used at a working distance of about 6.6 mm.

### 2.11. Environmental Scanning Electron Microscope

Protein adsorption and cell morphology on TNSs were studied using an environmental scanning electron microscope (ESEM, FEI Quanta 250 FEG, FEI Company) with a waterless cooling stage and a gaseous secondary electron detector. The proteins on the TNSs were imaged using a working distance of 6.1 mm, a pressure of 133 Pa, and an acceleration voltage of 5 keV.

Images of adherent cells were acquired at a chamber pressure of 431 Pa and a working distance of 6.0 mm to 6.3 mm. Depending on the sample, the accelerating potential of the electron beam was set between 5 kV and 8 kV. A humidity of 40% at a constant temperature of 8 ${}^{{}^{\circ}}$C was used. In order to highlight the proteins and cells on the TNSs, the captured images were post-processed with Affinity Designer for contrast and brightness, and both proteins and cells were stained.

### 2.12. Statistical Significance

Analysis of variance (ANOVA) was used to examine statistical significance with a self-written Python script [53,54,55,56,57]. The assumptions for ANOVA (normal distribution, similar variances, and independence of the data sets) were validated beforehand. One-way ANOVA was performed subsequently for each dataset. Statistical differences between two data points were computed using Tukey’s range test [60]. In the results, significant differences of p<0.05 are denoted with (*), while differences of p<0.01 are marked with (**).

## 3. Results

**Figure 1:**
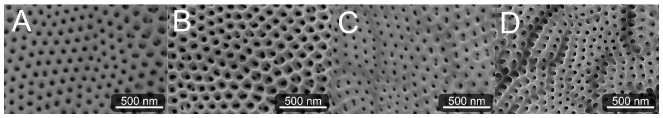
Scanning electron microscope (SEM) images of examined pristine TNSs (**A**) and TNSs implanted with 8 × 10${}^{14}\;\mathrm{ions}\cdot {\mathrm{cm}}^{-2}$ (**B**), 1 × 10${}^{16}\;\mathrm{ions}\cdot {\mathrm{cm}}^{-2}$ (**C**), and 2 × 10${}^{16}\;\mathrm{ions}\cdot {\mathrm{cm}}^{-2}$ (**D**). The inner tube radius decreases from (**A**–**D**) due to irradiation-induced viscous material flow, as specified in [50].

We prepared TNSs by electrochemical anodization in a self-organized growth process. To investigate the effect of carbon (C) implantation on protein adsorption and cellular response, TNSs were implanted with three different C fluences. Therefore, fluences of 8 ×10${}^{14}\;\mathrm{ions}\cdot {\mathrm{cm}}^{-2}$ (see Figure 1B) and 1 × 10${}^{16}\;\mathrm{ions}\cdot {\mathrm{cm}}^{-2}$ (see Figure 1C) with an ion energy of 60 keV yielded near-surface defects. Furthermore, 60 keV ions followed by 150 keV ions with 1 × 10${}^{16}\;\mathrm{ions}\cdot {\mathrm{cm}}^{-2}$ each were implanted to achieve 2 × 10${}^{16}\;\mathrm{ions}\cdot {\mathrm{cm}}^{-2}$ (see Figure 1D) and a more continuous implantation profile. We subsequently imaged the hexagonal pore-like surface morphology of the TNSs via scanning electron microscopy (SEM); see Figure 1. Image analysis of the inner tube radius yields tube shrinking of max. 58% from 29.8 ± 1.1 nm for pristine TNSs to 17.2 ± 0.9 nm for TNSs implanted with a fluence of 2 × 10${}^{16}\;\mathrm{ions}\cdot {\mathrm{cm}}^{-2}$. In fact, this tube shrinkage is connected to irradiation-induced viscous flow, as described by Kupferer et al. [49]. Apparently, stress cracks (dark gray) are present at higher fluences as well (Figure 1C,D) [50].

**Figure A1:**
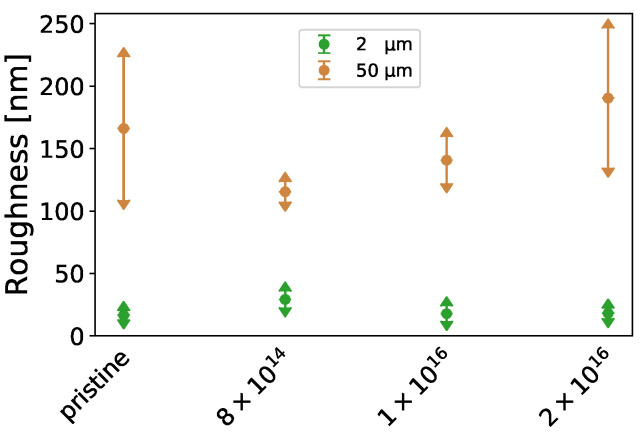
Determined mean square roughness for an area of 2 µm× 2 µm and 50 µm× 50 µm of the investigated TNSs.

**Figure A2:**
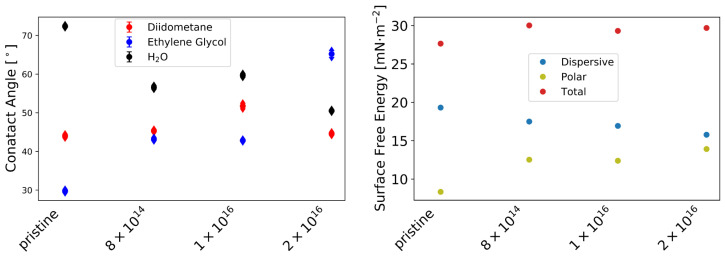
Contact angle measured with sessile drop method (**left**). Obtained dispersive, polar, and total surface free energy using Owens–Wendt–Rabel–Kaelble (OWRK) theory (**right**) [52].

Furthermore, the ζ-potential at pH=7 (see Table A1) increases with increasing carbon fluence from −26.94±1.60 mV for pristine TNSs to −12.20±1.40 mV for TNSs implanted with 2 × 10${}^{16}\;\mathrm{ions}\cdot {\mathrm{cm}}^{-2}$, while surface roughness (see Figure A1) remains relatively constant upon implantation. Contact angles measured via the sessile drop method decline from 72.37${}^{{}^{\circ}}\;\pm$ 0.24${}^{{}^{\circ}}$ to 50.50${}^{{}^{\circ}}\pm$ 0.22${}^{{}^{\circ}}$ by 30.22% for Milli-Q water, while they rise from 29.76${}^{{}^{\circ}}\;\pm$ 0.30${}^{{}^{\circ}}$ to 65.20${}^{{}^{\circ}}\;\pm$ 0.75${}^{{}^{\circ}}$ by 118.86% for ethylene glycol with increasing C implantation (see Figure A2, left). By employing the Owens–Wendt–Rabel–Kaelble (OWRK) theory, the surface free energy was derived from the contact angles. While the polar part of the surface free energy increases from 8.34 mN·m${}^{-2}$ to 13.91 mN·m${}^{-2}$ and the dispersive part decreases from 19.32 mN·m${}^{-2}$ to 15.18 mN·m${}^{-2}$ upon implantation, a slight increase in the total surface free energy is apparent (see Figure A2f, right). This change in surface free energy is connected to surface relaxation [61].

### 3.1. Protein Adsorption

**Figure 2:**
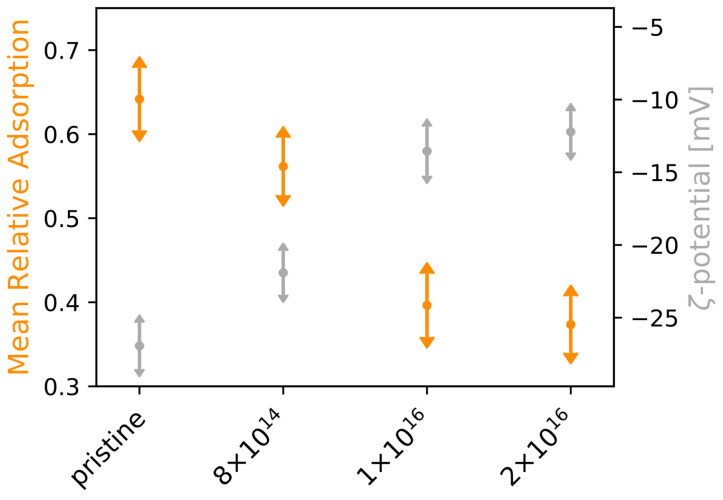
ζ-potential and mean relative laminin adsorption for examined TNSs. Protein adsorption experiments on TNSs were performed for four laminin concentrations (0.1, 0.5, 1.0, and 1.5 μg·cm${}^{-2}$). Ion fluences are given in ions·cm${}^{-2}$.

**Figure A3:**
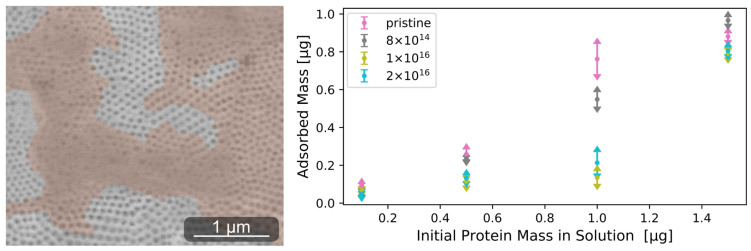
Environmental scanning electron microscope image of adsorbed rhodamine-labeled laminin cluster on pristine TNSs (**left**). Proteins were subsequently stained orange. Adsorbed amount of laminin determined after 30 min for four different initially applied protein concentrations (**right**). On all TNSs, increasing the protein mass in solution leads to a higher amount of adsorbed proteins. Elevating C fluence results in impediment to protein adsorption.

In order to investigate how the adsorption of laminin is affected by C implantation of TNSs, we applied four different concentrations of rhodamine-labeled laminin (0.1, 0.5, 1.0, 1.5 μg·cm${}^{-2}$) for 30 min at the surfaces and analyzed the scaffolds afterwards. With increasing initial protein concentration, we found that protein adsorption increases for all studied TNSs (see Figure A3, right). However, the implantation has a negative effect on adsorption, as it deteriorates with rising C fluence. Aiming for a physical explanation of the differences in protein adsorption, we determined the ζ-potential of the TNSs for different pH values. Interestingly, an indirect proportionality between ζ-potential under neutral condition and mean relative laminin adsorption becomes apparent (see Figure 2), i.e., while the mean relative laminin adsorption diminishes upon C implantation by 41.78%, the ζ-potential rises by 54.71%.

Because protein adsorption depends on the electrostatic characteristics of laminin, we further determined laminin’s ζ-potential in solution. For a neutral laminin solution of 20 μg$\cdot {\mathrm{mL}}^{-1}$, corresponding to an applied concentration of 2 μg·cm${}^{-2}$ in our experiments, we detected a ζ-potential of −23.98 ± 1.12 mV.

Time-dependent laminin adsorption was analyzed by fluorescence microscopy within 30 min. A droplet of laminin solution was pipetted onto the TNSs. Initially, the proteins exhibit very high diffusivity. However, proteins gradually accumulate at the edge of the droplet as well as on the TNSs surfaces. With increasing observation time, the mobility of proteins in the solution decreases and the protein complexes at the surface and droplet edge expand. These complexes are agglomerations of several proteins, as revealed by our dynamic light scattering measurements. Indeed, the hydrodynamic radius of laminin amounts to 24.16±8.00 nm, which is orders of magnitude smaller than the size of the detected complexes.

To further investigate the protein–TNS coupling, environmental scanning electron microscopy (ESEM) images of aggregated protein complexes on TNSs were acquired; see Figure A3, left. Larger protein complexes without preferential direction were detected on all TNSs.

### 3.2. Cell Viability and Cytotoxicity

**Figure 3:**
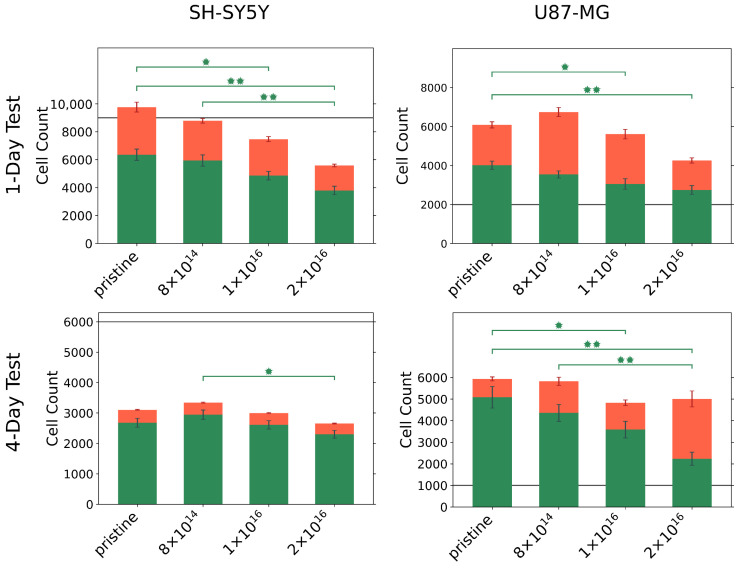
Number of viable (green) and dead (red) U87-MG and differentiated SH-SY5Y cells on pristine and C implanted TNSs after 1 and 4 day(s). Initially, 2000 U87-MG cells per square centimeter and 9000 differentiated SH-SY5Y cells per square centimeter were seeded for the one-day test (gray line). To perform the four-day test, 1000 U87-MG cells per square centimeter and 6000 differentiated SH-SY5Y cells per square centimeter were seeded (gray line). Significant differences of p<0.05 are marked with (*), and differences of p<0.01 are marked with (**). Ion fluences are given in ions·cm${}^{-2}$.

Because alterations in protein adsorption affect cell adhesion, and thus directly impact cell viability and toxicity, we were interested how C implantation affects cell viability and toxicity. For this purpose, we incubated U87-MG glial cells and differentiated SH-SY5Y neurons for 1 and 4 day(s), respectively, on TNSs, then analyzed the number of viable and dead cells; see Figure 3.

For all TNSs, we observed that U87-MG cells proliferate actively within one day, as the number of viable cells exceeds the number of initially seeded cells. However, the number of viable cells decreases with increasing C implantation. Similar behavior is found during the four-day test; the cell number of seeded U87-MG cells increases by a factor of six compared to the initial cell number. With rising carbon fluence, a significant decline in viable cells can be recognized, with only a slight elevation in the number of dead cells. Accordingly, the ratio of viable to dead cells changes from 86%:14% for the pristine TNSs to 45%:55% for the TNSs implanted with 2 $\times \;10^{16}\;\mathrm{ions}\cdot {\mathrm{cm}}^{-2}$.

Contrasting these results, differentiated SH-SY5Y cells grew differently on the TNSs; with a lower number of detected cells compared to the number of initially seeded cells. In particular, approximately 50% of the initially seeded cells could be detected after four days. A significant decrease in the number of viable cells with increasing C fluence was apparent for SH-SY5Y cells after both test periods. However, we highlight the high cell viability (>85%) of the detected cells for the four-day test, which did not appear to be influenced by carbon implantation.

### 3.3. Cell Morphology

**Figure 4:**
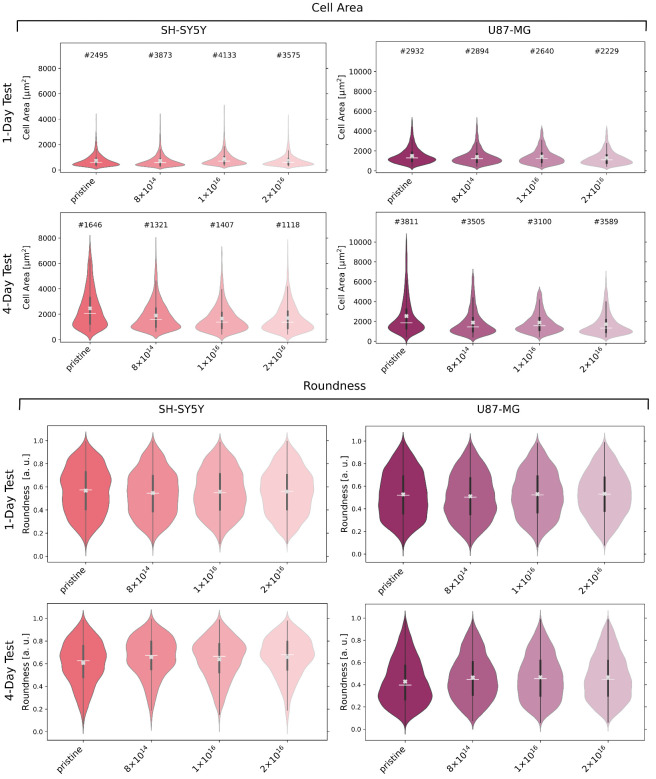
Cell area and cell roundness of U87-MG and differentiated SH-SY5Y cells after incubation of one and four days(s), respectively, on examined TNSs. Analyzed cell count (#), mean (white line), median (white cross), and interquartile range (dark gray) of the data distribution of detected cells. Ion fluences are given in ions·cm${}^{-2}$.

In order to explore the effect of C implantation on cell morphology, we stained and detected cells after one and four day(s) to analyze cell area and roundness (see Figure 4). For U87-MG cells and differentiated SH-SY5Y cells, no significant relation between cell area and carbon fluence is evident after one day. However, U87-MG cells are about twice as large as SH-SY5Y cells. A decrease in cell area becomes apparent after four days for both cell types with increasing carbon fluence. In detail, at median, the cell area of U87-MG cells for the TNSs with a fluence of 2 $\times \;10^{16}\;\mathrm{ions}\cdot {\mathrm{cm}}^{-2}$ is 28% less than for the pristine TNSs. This difference in median cell area is more prominent for SH-SY5Y cells, with a percentage decrease of 33%.

The roundness of U87-MG, as well as of SH-SY5Y cells after one day, appears to be independent of C implantation, as the median cell roundness does not significantly differ for all TNSs. After four days, the roundness of the U87-MG cells decreases on all TNSs, i.e., cells are more elongated. However, cells on the TNSs implanted with 2 $\times \;10^{16}\;\mathrm{ions}\cdot {\mathrm{cm}}^{-2}$ are 12% more round than cells on the pristine TNSs.

Slightly different behavior can be observed for the SH-SY5Y cells: for the studied TNSs, the roundness of the cells rises after four days. Compared to the one-day test, an increase in roundness with C implantation is observable, just as detected for U87-MG cells. However, the roundness increases by only 8% for the TNSs with 2 $\times \;10^{16}\;\mathrm{ions}\cdot {\mathrm{cm}}^{-2}$ as compared to the pristine TNSs.

### 3.4. Cell Adhesion

**Figure 5:**
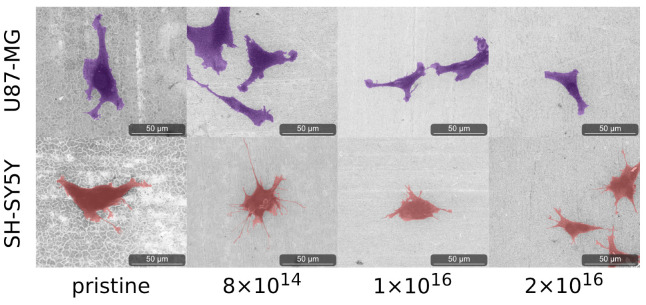
Images of analyzed cells acquired with ESEM after four days on the examined TNSs. To highlight the cells, we stained U87-MG purple and SH-SY5Y pink. Ion fluences are given in ions·cm${}^{-2}$.

**Figure A4:**
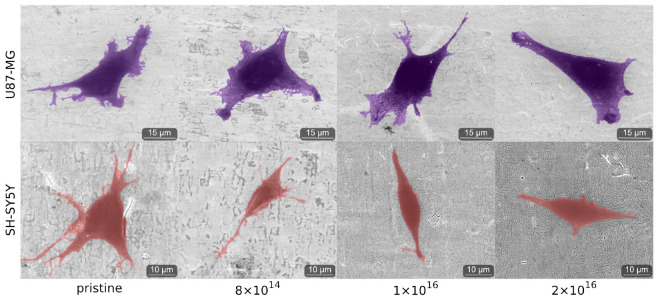
Analyzed extensively expanded U87-MG and SH-SY5Y cells after one day on examined TNSs. U87-MG cells are stained purple and SH-SY5Y cells are labeled pink. Increasing C implantation leads to a decline in cell protrusions of U87-MG cells. SH-SY5Y cells exhibit a decrease in the number and length of cell fibers upon rising implantation of TNSs.

As C implantation affects cell morphology, cells of both cell lines were analyzed by environmental scanning electron microscope (ESEM) after one and four days. After one day, qualitative differences in cell morphology associated with C implantation of TNSs were not detected for either differentiated SH-SY5Y cells or for U87-MG cells; see Figure A4. However, the cell morphology of both U87-MG cells and SH-SY5Y in the four-day tests changes with respect to different C implantation of TNSs; see Figure 5.

We observed elongated U87-MG cells on all TNSs. Additionally, their nucleus is visible, as it is located in the region of the cells that is darker due to the higher amount of carbon. Cells on pristine TNSs and TNSs with a fluence of 8 $\times \;10^{14}\;\mathrm{ions}\cdot {\mathrm{cm}}^{-2}$ show rough cell edges, as the cells form many cell protrusions. With increasing C implantation, cell protrusions seem to diminish and the cell edges appear much smoother. Additionally, the cell area decreases qualitatively with rising ion implantation, as cells appear slightly smaller.

SH-SY5Y cells exhibit similar behavior as U87-MG cells, adhering and expanding on all TNSs. Cells on pristine TNSs and TNSs with fluence of 8$\times 10^{14}\;\mathrm{ions}\cdot {\mathrm{cm}}^{-2}$ expand extensively over the surfaces, developing multiple long cell fibers (presumably dendrites). With increasing carbon implantation cells no longer extend as much, and the number and length of the cell fibers are considerably smaller. However, the cell protrusions (as measured perpendicular to the direction of cell spreading) appear thicker with higher implanted fluences. On pristine and 8 $\times \;10^{14}\;\mathrm{ions}\cdot {\mathrm{cm}}^{-2}$-implanted TNS, we observe thin and long dendrites, while on 2 $\times \;10^{16}\;\mathrm{ions}\cdot {\mathrm{cm}}^{-2}$ the protrusions are shorter and much more expanded.

## 4. Discussion

Ion implantation is a well-established method in tuning material and surface characteristics [62,63,64]. Indeed, Kupferer et al. have recently reported that low-energy low-fluence ion implantation of TNSs not only promotes smoothing and surface relaxation, it is further connected to a considerable change in chemical composition and electrical conductivity [49,61]. In particular, sensible neuronal tissues and cells perceive modest changes in their culture substrates and adjust their proliferation and mechanotransduction accordingly. Hence, we investigated the influence of C implantation on protein adsorption and cell viability as well as on cell morphology to assess biocompatibility and the general suitability of C implanted TNSs for novel neural implant applications. To this end, we analyzed the adsorption of laminin on TNSs under neutral conditions for different protein concentrations. In the time-dependent experiments, we found that laminin adsorption on TNSs occurred within 30 min, as reported by Giamblanco et al. [65]. Our results contrast the findings of Lin et al. [66], who detected a time-delayed adsorption kinetic of laminin. Furthermore, we observe that smaller protein complexes adsorb to the surfaces first, then grow over time. Related theoretical findings have been reported by Mulheran et al. using Monte Carlo simulations [67], and Rabe et al. [68] and Pellenc et al. [69] obtained similar experimental results. However, the adsorption rate was slightly higher in our experiments. It is possibly that different environmental conditions such as temperature or protein–protein interactions are responsible for this. Indeed, our obtained hydrodynamic radius is in the range of the value of 20.6±0.5 nm [70] from the published literature. This indicates that the detected protein complexes are magnitudes larger than individual proteins. Additionally, we observed a temporal aggregation of larger protein complexes at the droplet edge, as reported by Pal et al. [71] and Tarafdar et al. [72], due to the evaporation being the highest at the vapor–liquid–solid interface. More particles move to this interface, resulting in a higher adsorption rate compared to the center of the droplet.

Our quantitative protein adsorption studies indicate that the adsorbed protein mass on the surfaces rises with increasing protein concentration for all investigated TNSs. Similar findings have been presented by Young et al. [73], Chen et al. [74], and Fujita et al. [75]. In addition, we discovered that C implantation negatively affects protein adsorption, as protein adsorption decreases with increasing C fluence. Because protein adsorption on surfaces is mainly caused by electrostatic protein–surface interactions, we suggest that surface charges change upon implantation, as demonstrated by our ζ-potential measurements. With increasing C fluence, the ζ-potential rises and the mean relative protein adsorption diminishes. A similar relation between ζ-potential and the adsorbed amount of laminin has been reported by Lin et al. [66]. However, their determined ζ-potential was stated to be +6.2±0.9 mV, which drastically deviates from our results. We further observed a change of surface free energy upon implantation, as shown by Kupferer et al. [61]. Already in 1988, Young et al. [73] found that proteins strongly adsorb to surfaces with a high dispersive and low polar part of the surface free energy. This matches our results, as the dispersive part of the surface free energy decreases with rising ion fluence while the polar part increases. However, an influence of surface roughness on protein adsorption, as reported by Giamblanco et al., was not observed [65].

Interestingly, the inner diameter of the nanotubes shrinks with increasing ion fluence. Consequently, we assume that smaller nanotube diameters might deteriorate protein adsorption due to the reduced accessible surface area. We claim that formed protein complexes are rather unlikely to enter the inside of the nanotubes, contrary to the results stated by Kulkarni et al. [76] and Rahman et al. [77].

To investigate the effect of C implanted TNSs on the adhesion and proliferation of differentiated SH-SY5Y neuroblastoma cells and U87-MG gliablastoma cells, we incubated cells for one and four days on the TNSs and analyzed them accordingly. After one day, we detected large numbers of viable cells and only a minority of dead cells for both cell lines. Together with the vigorous proliferation behavior of the U87-MG cells and the high cell viability of the SH-SY5Y cells after four days, this demonstrates that all examined TNSs act as excellent support scaffolds for our investigated cells. However, we observed a significant decrease in cell viability with increasing C fluence, especially during the four-day tests, implying that implantation unfavorably effects cellular behavior. Looking for an explanation, we concentrate on material characteristics that might alter cell viability. Yoon et al. [78] and Brunetti et al. [79] reported that cell viability of SH-SY5Y cells depends on surface roughness. Similarly, Abend et al. [80] demonstrated that roughness affects proliferation of U87-MG cells. However, in our experiments we were not able to identify a distinct correlation between cell viability and TNS surface roughness, similar to Hallab et al. [81]. We assume that the decrease in the number of viable cells might be induced by changes in surface charge or altered composition. Indeed, changes in composition affected cell proliferation, as reported by Abend et al. for indium tin oxide (ITO), gold, nanocolumnar titanium nitride (nanoTiN), and titanium nitride (TiN) surfaces [80]. In particular, U87-MG cells proliferated very strongly within three days on gold, nanoTiN, and TiN surfaces. Strong proliferation of differentiated SH-SY5Y cells was observed on titanium surfaces, which contrasts with both our findings and the results of Yoon et al. [78].

We hypothesize that the decreasing viability of cells upon implantation in our experiments can be explained by the change in the surface free energy with increasing C fluence. Specifically, we suggest that proteins from the cell culture medium favorably adsorb to pristine TNSs because of the high dispersive and low polar components of the surface free energy. This is in line with the high amount of adsorbed laminin, and might favor (early) cell adhesion, as observed by Weidt et al. [37]. The higher the implanted C fluence, the lower the dispersive component and the higher the polar component of the surface free energy of the TNSs, resulting in a deterioration of protein binding and cell adhesion.

It is commonly known that tube diameter influences cell viability. For instance, Tian et al. demonstrated that the cell viability of U87-MG cells after 24 h is highest on TNSs with a tube diameter of 20 nm [82]. They further reported that a tube diameter increase to 120 nm resulted in a decrease in viability by about 50%. Related to our findings, the high rate of dead U87-MG cells after one day suggests an unfavorably chosen tube diameter. Similar results were reported by Yang et al. [83]; C6 glial cells were incubated on TNSs with different tube diameters for 24 h. On the TNSs with 15 nm tube diameter 115% of the initially seeded cells were viable, while on the TNSs with 30 nm tube diameter only 85% were viable. They concluded that cell proliferation is particularly favored at small tube diameters, similar to Tian et al. In contrast with these results, we observed that cell viability dropped with decreasing tube diameter, viz., with increasing C fluence, suggesting that this effect is superimposed with the change of surface free energy.

We propose that the increase in electrical conductivity is responsible for the decrease in cell viability with increasing implanted C fluences. Choi et al. demonstrated that cell viability of SF295 gliablastoma cells declines with increasing electrical conductivity of ZnO thin films [84]. They postulated that cells on conductive surfaces may adhere strongly to the surface due to the increased electrostatic interaction. No focal adhesion complexes are formed in this process, resulting in cessation of cell proliferation. However, our studied TNSs are not metallic conductors; hence, proliferation of U87-MG cells is observed.

Moreover, we analyzed cell morphology and found similar trends as for cell viability. After one day, the mean average area of cells on all investigated TNSs did not significantly differ. However, U87-MG cells are approximately twice as large as SH-SY5Y cells. After four days, we observed a correlation between carbon fluence and cell size for both cell lines. With higher carbon fluence, the cell area decreases, i.e., cells on the TNSs do not expand as much, as depicted in the ESEM images. In addition, SH-SY5Y cells form fewer and shorter dendrites with increasing C fluence. Similar results were obtained for U87-MG cells. While cells form protrusions and are more elongated on pristine TNSs, the cells exhibit a rounder morphology on TNSs implanted with higher C fluences. Interestingly, we found that the cell areas of both cell lines on TNSs are approximately one order of magnitude higher compared to cells on TiN and nanocolumnar TiN, as reported by Abend et al. [85].

Because we focused on the response of several C fluences implanted in TNSs, we believe that fine-tuning of TNSs by modulating tube diameter and surface functionalization has great potential. In particular, the initially detected functional groups on the TNSs surface change upon the use of cell culture medium with serum consisting of salts, sugars, and several proteins. Consequently, competing protein adsorption kinetics are at play, and the variety of predominant functional groups impacts initial cell adhesion. To keep the system in our study as clear and concise as possible, we focused on laminin and illustrated the fundamental principles of laminin adsorption with concentrations resembling in vivo conditions and human brain cell performance on implanted TNSs.

Our prospective experiments will focus on the adsorption kinetics of a protein mixture in which a competing adsorption of several proteins takes place. Targeting the intended later use as an neuronal implant material, we intend to assess a co-culture of neurons and glial cells or adult brain tissue culture on TNSs with different tube diameters and morphologies.

## 5. Conclusions

In conclusion, while laminin is adsorbed on all TNSs, the absorbed amount decreases with increasing C implantation. After four days of incubation time, glial U87-MG cells proliferate, and highly viable neuronal SH-SY5Y cells can be detected.

The cell morphology indicates that C ion implantation does not influence the biocompatibility of the TNSs, as cells stretch extensively on all investigated scaffolds. Interestingly, for high C fluences, SH-SY5Y cells revealed high cell viability, while U87-MG cells did not proliferate as much as on pristine TNSs. Hence, we postulate that implanted TNSs may be auspicious candidates for brain–machine interfaces in the future. Inflammatory responses such as glial scarring, which occur in contact with current in vivo implants, might be reduced. Furthermore, the low cell toxicity indicates that implanted TNSs provide an exceptional basis for novel MEAs with regard to long-term stability. In order to address these questions in more detail, our future studies will focus on the interaction of primary neurons and glial cells with implanted TNSs, as well as cell behavior within entire brain slices cultured on TNSs.

Because the surface characteristics of implanted TNSs were rather unspecific and not specifically optimized for individual cell types, we propose a further fine-tuning by adjusting nanotube diameters, surface functionalization, etc. Furthermore, as tissues may respond differently to the C implanted TNSs compared to cells, culturing neuronal tissue on implanted TNSs would be convenient for prospective experiments. In this way, degenerative effects and rejection reactions could be analyzed. Prospectively, C implanted TNSs might pave the way to a new class of biocompatible, conductive, and highly tailorable materials for brain–machine interfaces.

## Data Availability

Data available on request from the authors.

## Appendix A. Results and Discussion

### Appendix A.1. Surface Characterization

**Table A1:** ζ-potential of TNSs at pH=7.

Fluence [ions·cm${}^{-2}$]	ζ-Potential [mV]
pristine	−26.94±1.60
8 ×10${}^{14}$	−21.91±1.52
1 ×10${}^{16}$	−13.55±1.70
2 ×10${}^{16}$	−12.2±1.43

## Appendix B. Sample Cleaning

After experiments, all samples were cleaned for 24 h in a ThermoMixer (Eppendorf AG) at a temperature of 55 ${}^{{}^{\circ}}$C with lysis buffer. The buffer was prepared using 10 mL 100 mM Tris-HCl (Cat. No. 1185-53-1, Carl Roth GmbH and Co. KG) at pH 8.5, 1 mL 5 mM ethylenediaminetetraacetic acid (Cat. No. L2143, Biochrom GmbH, Berlin, Germany), 1 mL 0.2% sodium *n*–dodecyl sulfate (Cat. No. 1125330050, Sigma-Aldrich Chemie GmbH), 4 mL 200 mM sodium chloride (Cat. No. S9888-1KG, Sigma-Aldrich Chemie GmbH), 50 mg Proteinase K (Cat. No. P2308-1G, Sigma-Aldrich Chemie GmbH), and 84 mL Milli-Q water. Finally, TNSs were sonicated for 1 min, cleaned with a strong stream of Milli-Q water, and subsequently dried under nitrogen flow.
